# Amplification of telomerase (*hTERT*) gene is a poor prognostic marker in non-small-cell lung cancer

**DOI:** 10.1038/sj.bjc.6603110

**Published:** 2006-04-25

**Authors:** C-Q Zhu, J-C Cutz, N Liu, D Lau, F A Shepherd, J A Squire, M-S Tsao

**Affiliations:** 1 Division of Applied Molecular Oncology, Ontario Cancer Institute, Ontario, Toranto, Canada; 2 Department of Pathology, Princess Margaret Hospital, University Health Network, Toronto, Ontario, Canada; 3 Division of Hematology and Medical Oncology, Princess Margaret Hospital, University Health Network, Toronto, Ontario, Canada; 4 Department of Medicine, University of Toronto, Toronto, Ontario, Canada M5G 2M9; 5 Department of Laboratory Medicine and Pathobiology, University of Toronto, Toronto, Ontario, Canada M5G 2M9; 6 Department of Medical Biophysics, University of Toronto, Toronto, Ontario, Canada M5G 2M9

**Keywords:** h*TERT*, telomerase, laser-captured microdissection, QPCR, FISH

## Abstract

Telomerase reactivation is a hallmark of human carcinogenesis. Increased telomerase activity may result from gene amplification and/or overexpression. This study evaluates the prognostic value of h*TERT* gene amplification and mRNA overexpression in 144 resectable non-small-cell lung cancer (NSCLC) specimens. The h*TERT* gene copy number was assessed by quantitative polymerase chain reaction (qPCR) on laser-capture microdissected tumour cells of 81 tumours, and by fluorescence *in situ* hybridisation (FISH) on a subset of 59 tumours. h*TERT* mRNA level was determined by reverse transcription (RT)–qPCR in 130 tumours. In total, 57% of (46 out of 81) primary NSCLC specimens demonstrated h*TERT* amplification, which was significantly more common (*P*<0.001) in adenocarcinoma (30 out of 40) than in squamous cell carcinoma (13 out of 37). The h*TERT* mRNA overexpression was noted in 74% (94 out of 130) of tumours; it was more frequent in squamous cell than in adenocarcinoma (87 *vs* 68%, *P*=0.03). Overexpression was significantly associated with amplification (*P*=0.03), especially in adenocarcinoma. The h*TERT* gene amplification was prognostic for shorter recurrence-free survival (hazard ratio=2.16, *P*=0.03). These data indicate that gene amplification is an important mechanism for h*TERT* overexpression in lung adenocarcinoma and is an independent poor prognostic marker for disease-free survival in NSCLC.

Telomerase is a ribonucleoprotein reverse transcriptase complex containing an RNA subunit h*TERC* and a protein catalytic subunit h*TERT* (Nakamura *et al*, 1997). The h*TERC* RNA is expressed universally in eukaryotic cells and h*TERT* expression has been correlated with activation of the telomerase complex (Nakamura *et al*, 1997; Kolquist *et al*, 1998; Arinaga *et al*, 2000). Telomerase activity is absent in most human adult somatic cells. With continuous cell proliferation, there is a progressive loss of telomeric DNA that ultimately may trigger replicative senescence. The forced expression of h*TERT* cDNA in normal human cells has led to extension of the replicative lifespan (Vaziri and Benchimol, 1998). An alternative mechanism for cellular immortalisation is by the telomerase-independent ALT pathway (Newbold, 2002). Activations of the h*TERT* or ALT pathways are obligate for senescence bypass and for neoplastic transformation of normal cells (Newbold, 2002). Telomerase activity and/or h*TERT* expression are increased in cancers and are prognostic factors in various cancer types (Harada *et al*, 1999; Bieche *et al*, 2000; Lee *et al*, 2001; Marchetti *et al*, 2002; Wang *et al*, 2002; Fujita *et al*, 2003; Krams *et al*, 2003; Ohali *et al*, 2003; Tchirkov *et al*, 2003; Lantuejoul *et al*, 2004). However, the clinical impact of h*TERT* expression or activity in non-small-cell lung cancer (NSCLC) remains controversial (Albanell *et al*, 1997; Taga *et al*, 1999; Hirashima *et al*, 2000; Komiya *et al*, 2000; Toomey *et al*, 2001; Marchetti *et al*, 2002; Wang *et al*, 2002; Fujita *et al*, 2003; Wu *et al*, 2003; Hsu *et al*, 2004; Lantuejoul *et al*, 2004). The clinical significance of increased h*TERT* gene copy number has not been investigated.

Multiple mechanisms may regulate h*TERT* expression and activity. There is considerable evidence that transcriptional activation plays a major role in regulating h*TERT* mRNA expression (Ducrest *et al*, 2002), and the latter is correlated with telomerase activity (Arinaga *et al*, 2000; Marchetti *et al*, 2002; Saretzki *et al*, 2002). However, post-translational modifications may also contribute to the regulation of h*TERT* activity (Kang *et al*, 1999). Increased expression of h*TERT* resulting from gene amplification was recently reported in embryonal brain tumours (Fan *et al*, 2003) and cervical carcinoma (Zhang *et al*, 2002). The h*TERT* gene is located on chromosome 5p15, a chromosomal arm that is commonly overepresented or amplified in lung cancer (Luk *et al*, 2001). Amplification of the h*TERT* gene and a concomitant increase in telomerase activity has been reported in lung cancer cell lines and other cancer types (Zhang *et al*, 2000; Saretzki *et al*, 2002). In this study, we have investigated the frequency and prognostic significance of h*TERT* gene amplification and overexpression in NSCLC.

## MATERIALS AND METHODS

### Patients and clinical samples

Patients included in this study had undergone lobectomy or pneumonectomy for resection of their primary lung cancer but had not received prior radiation or chemotherapy. Altogether, 169 tissue samples from 144 patients were used; these included 144 tumours and a corresponding subset of 30 non-neoplastic lung tissues. The latter were used to define the normal ranges for h*TERT* mRNA expression levels and gene copy number. Tissues were collected within 30 min after resection, snap-frozen and stored in liquid nitrogen until used; all were verified by histopathology. The collection of tissue and clinical and follow-up data was carried out in accordance with guidelines established by the Research Ethics Board (REB) of the University Health Network (UHN), which also approved this study.

### DNA isolation and laser-captured microdissection

DNA was isolated from tumour cells micro-dissected using the Arcturus Pixcell II (Mountain View, CA, USA) laser capture microdissection (LCM) system. This includes 40 adenocarcinomas (ADC), 37 squamous cell carcinomas (SQCC), three adenosquamous carcinomas (ADSQC) and one large cell carcinoma (LCC). In addition, DNA was also extracted from 19 non-neoplastic lung samples. The tumour cells micro-dissected using LCM system were incubated in DNA extraction buffer containing 50 mM KCl, 10 mM Tris-HCl (pH 8.3), 0.1 mg ml^−1^ gelatin, 0.45% Nonidet P-40, 0.45% Tween 20 and 0.4 mg ml^−1^ proteinase K. DNA was subsequently extracted by the phenol–chloroform method (Zhu *et al*, 2004).

### Quantitative polymerase chain reaction

The quantitative polymerase chain reaction (qPCR) was performed using the SYBR Green technique in an ABI Prism 7700 sequence Detection System (Applied Biosystem, Foster City, CA, USA). The primer sequences were: h*TERT* sense 5′-taa aat tat cca cat ggc tca cgt-3′, antisense 5′-ctt ggg aac cag gac aaa gg-3′; *PIK3R1* sense 5′-atc tgc cac tgg ctt ccc tt-3′, antisense 5′-cag tct ttc cct gat cat tga acc-3′. The PCR conditions were optimized as reported (Zhu *et al*, 2004). The *PIK3R1* (5q13.1) gene is used as the reference nonamplified gene in NSCLC (Massion *et al*, 2002), and the h*TERT* gene copy number was estimated using comparative CT method. DNA from normal male lymphocyte (Novagen, San Diego, CA, USA) was used as the reference DNA. With this method, samples with normal copy number (disomy at both loci) or balanced polysomy (increased but equal copy number of both the reference and h*TERT* loci) will have an h*TERT*/*PIK3R1* ratio of 1. Copy number values above or below the 2 standard deviations (s.d.) of mean of normal lung tissues values were designated as amplified or loss of the h*TERT* gene copy. Tumours with h*TERT* amount within the 2 s.d. of mean of normal lung tissues were classified as showing nonamplified samples.

### Fluorescence *in situ* hybridisation

Archival paraffin blocks of 59 tumours that had been studied by qPCR were retrieved for fluorescence *in situ* hybridisation (FISH) analysis. Sections (4 *μ*m) were mounted on positively charged slides and baked flat for 12–16 h at 56°C. Slides were dewaxed in three changes of xylene for 10 min each, followed by two changes in 100% ethanol for 5 min each. After air-drying, slides were treated in 2 × SSC for 20 min at 75°C, then for 5 min at room temperature (RT). The sections were then treated with 0.25 mg ml^−1^ proteinase K (Roche, Laval, QC, Canada) in 2 × SSC at 45°C for 20 min, followed by washing in 2 × SSC at RT for 5 min and serial dehydration through 70, 90 and 100% ethanol, and then left to air dry.

The h*TERT*/5q dual-colour FISH probe cocktail (Qbiogene, Montreal, QC, Canada) was applied at 0.02–0.06 *μ*l mm^−2^ and sealed with rubber cement. The probe and target DNA were codenatured by heating to 80°C for 10 min in a Hybrite slide incubator (Vysis/Abbott Laboratories, Markham, ON, Canada). Hybridisation was for 16–20 h at 37°C in a moist light-sealed chamber in a dry oven. The slides were washed in two changes of 2 × SSC with 0.1% SDS at 45°C for 5 min each, followed by 5 min in 2 × SSC at RT. Slides were partially air-dried and 20–30 *μ*l of DAPI mounting medium with antifade (Vector Labs, Burlingame, CA, USA) was applied, then cover slipped without sealing. Slides were stored in the dark at −20°C prior to imaging.

The FISH images were captured using the AxioImager system (Zeiss, Göttingen, Germany) with Z-stacking capabilities. Tumour cell nuclei identified using a DAPI filter and Z-stacked three-channel colour images (DAPI, FITC and Rhodamine/Cy3) were captured at × 63 or × 100 under oil immersion. Intact, nonoverlapping tumour cell nuclei (minimum 50 per case) without juxtaposed FISH signals were scored for the number of green (5p15.33) h*TERT* locus and red (5q31) control signals. The surrounding nontumour cells provide baseline estimation of the normal FISH signals (two green and two red signals). For survival analysis, high gene copy number cases included tumours with high polysomy (⩾4 h*TERT* gene copy in more than 40% of the tumour cells) or amplification (presence of tight h*TERT* gene clusters and a ratio of h*TERT* to chromosome of ⩾2 or ⩾15 copies of gene per cell in ⩾10% of analysed tumour cells), as defined by Cappuzzo *et al* (2005) for their study of the role of epidermal growth factor receptor (EGFR) gene copy number in EGFR inhibitor therapy.

### Reverse transcription–qPCR

The mRNA expression was assayed using reverse transcription (RT)–qPCR on total RNA of 130 primary NSCLC and 18 corresponding non-neoplastic lung tissues using the ABI PRISM 7700 Sequence Detection System (Zhu *et al*, 2004). Total cellular RNA was isolated from the frozen tissues, as previously described (Tsao *et al*, 1998) and purified by the RNeasy Mini kit (Qiagen Inc., Mississauga, ON, Canada). The quality of the RNA preparations was confirmed by the Agilent Bioanalyzer (Agilent Technologies, Palo Alto, CA, USA). In total, 5 *μ*g of RNA was reverse transcribed using the Taqman reverse transcription reagent (Applied Biosystems, Branchburg, NJ, USA) in 100 *μ*l reaction solution according to the manufacturer's instruction. After appropriate dilution, duplicate of 10 ng of cDNA was used as template for qPCR analysis of each sample. Primers were designed to span two adjacent exons to avoid amplification of contaminating genomic DNA sequences. The primers for h*TERT* were: sense 5′-cgtcgagctgctcaggtctt-3′, antisense 5′-agt gctgtctgattccaatgctt-3′. The ΔCT method was used to normalize the sample-to-sample variation in RNA/cDNA quantity using the 18s ribosomal RNA as the housekeeping gene (Zhu *et al*, 2004).

### Statistical analysis

The Spearman correlation, *χ*^2^ tests or Fisher's exact test were used appropriately to assess association within and between molecular indices and the pathological or clinical factors. The end points for analyses were overall survival (from date of surgery to date of death) and recurrence-free survival (from date of surgery to date of recurrence). Cox proportional hazards regression was used in univariate and multivariate analyses. For Kaplan–Meier analysis, gene copy number and mRNA expression level were dichotomized using the upper limits of 95% confidence interval (95% CI) (mean+2 s.d.) for normal samples into nonamplified *vs* amplified or normal expression *vs* overexpression groups. Kaplan–Meier analysis estimates the survival of patient groups, and significant differences were determined by the log-rank test.

## RESULTS

### Patient characteristics

Table 1 shows the demographics of patients in the studies of h*TERT* gene copy assessment by qPCR (*n*=81) or FISH (*n*=59), and h*TERT* mRNA expression (*n*=130) by RT–qPCR. There were no significant differences in the age, gender, stage and tumour differentiation grade among the three groups, but the mRNA expression study included more ADC patients. More than 90% of patients were stage I–II. The median follow-up was 3.19 (0.24–7.93) years, and 11 patients died without a relapse.

### hTERT gene amplification

**Figure 1:**
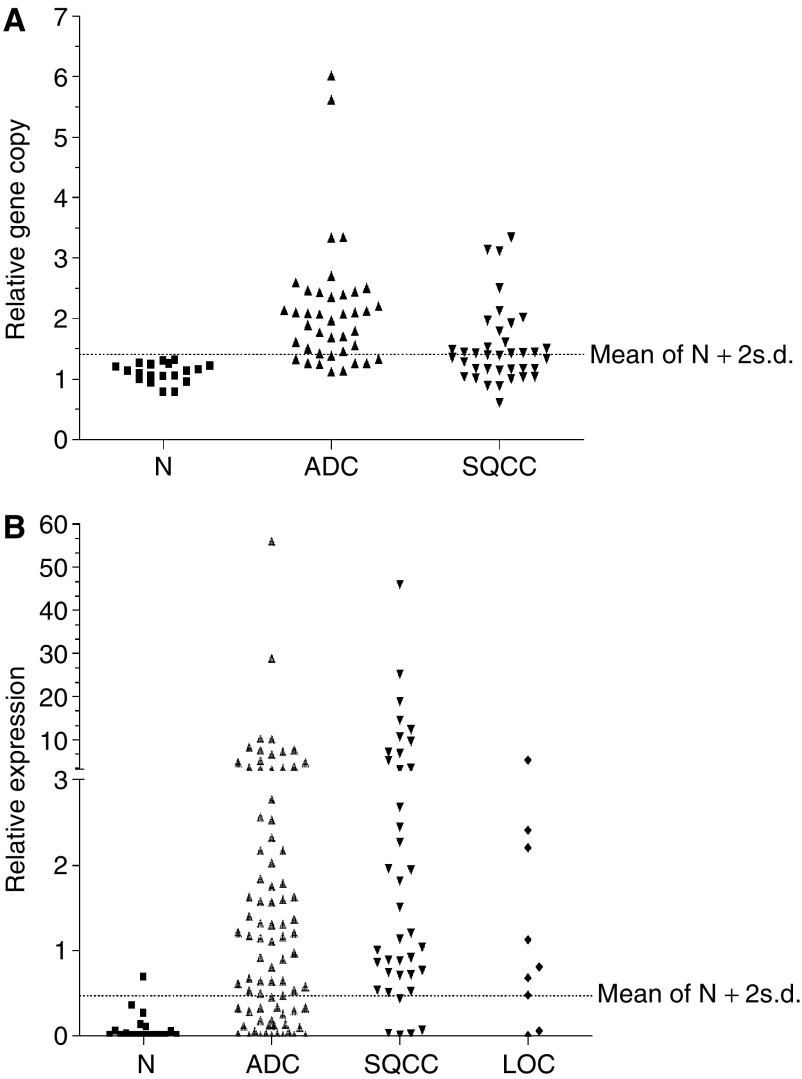
The distribution of h*TERT* gene copy number and mRNA expression levels according to tissue and tumour type. N, normal lung tissue; ADC, adenocarcinoma, SQCC, squamous cell carcinoma; LCC, large-cell lung carcinoma.

**Figure 2:**
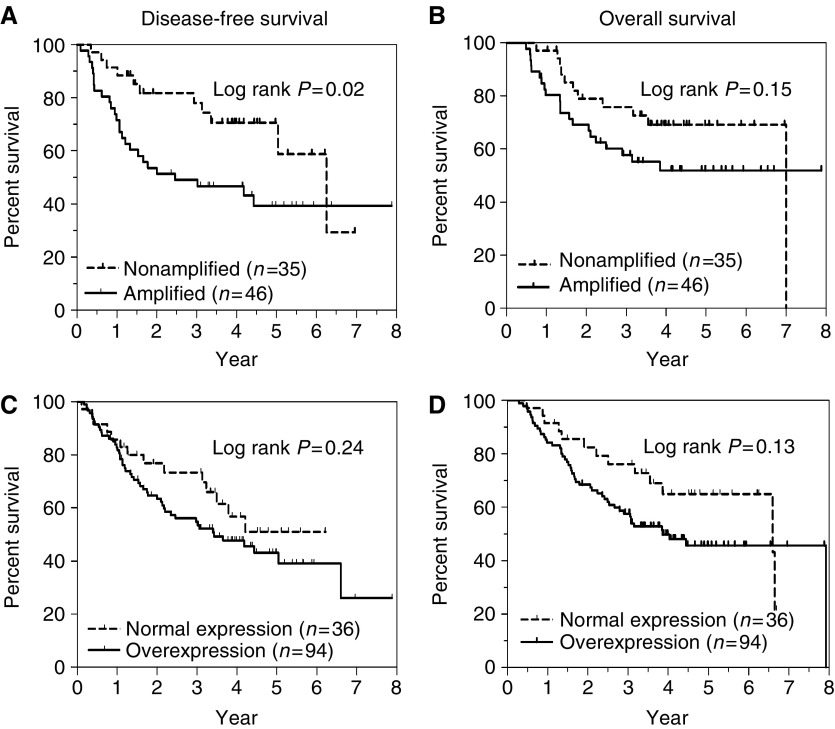
Kaplan–Meier survival plots. (**A**) Recurrence-free survival (RFS) according to h*TERT* gene copy. (**B**) Overall survival (OS) according to h*TERT* gene copy. (**C**) RFS according to expression levels of h*TERT* mRNA expression levels. (**D**) Overall survival according to h*TERT* mRNA expression levels.

Figure 1A shows the distribution of relative gene copy of h*TERT* in normal and NSCLC. Using the upper limit of 95% CI (mean+2 s.d.) for normal samples as cutoff, h*TERT* gene amplification was found in 57% (46 of 81) of NSCLC patients. Gene copy loss was not observed. Amplification was more common in ADC compared to SQCC (Figure 1A), but was not correlated with tumour stage or differentiation grade (Table 2). The Kaplan–Meier survival estimation showed that patients with h*TERT* amplification by qPCR had poorer recurrence-free survival (log rank test *P*=0.02, Figure 2A). An analysis using 2 × mean of normal+2 s.d. as the cutoff to identify highly amplified patients showed statistically not significant separation of the survival curves of amplified *vs* unamplified patients, but further analysis showed that patients with gene copy changes between ⩾mean of normal+2 s.d. and <2 × mean of normal+2 s.d. showed similar survival outcome as the highly amplified (⩾mean of normal+2 s.d.) group, indicating that low amplification patients also experienced poorer survival outcome (Supplementary Figure 1). A similar trend of poorer overall survival for patients with amplified h*TERT* gene was noted, but this did not reach significance (log rank test *P*=0.15, Figure 2B).

Cox proportional hazards regression also showed a significant association of h*TERT* amplification with increased risk for death from recurrence (hazard ratio (HR) 2.16, 95% CI 1.07–4.37; *P*=0.03), but the correlation with poorer overall survival did not reach significance (HR 1.70, 95%CI 0.82–3.52; *P*=0.16). The h*TERT* amplification remained a significant prognostic marker for shorter recurrence-free survival (HR 2.06, 95%CI 1.01–4.2; *P*=0.05) after adjusting for the patient age, tumour stage and differentiation grade.

Because there was a high frequency (38%) of patients who were lost to follow-up at greater than 3 years after surgery, the 3-year survival rates were also estimated (Table 3). h*TERT* amplification was significantly associated with poorer recurrence-free survival (HR 2.96, 95% CI 1.27–6.90, *P*=0.01) and overall survival (HR 2.04, 95% CI 0.89–4.66, *P*=0.09) at 3 years. Multivariate analysis adjusting for patient age, tumour stage and differentiation grade confirmed that amplification was an independent prognostic marker for recurrence-free survival (HR 2.97, 95% CI 1.26–6.99, *P*=0.01; Table 3).

### Validation of qPCR data with FISH

**Figure 3:**
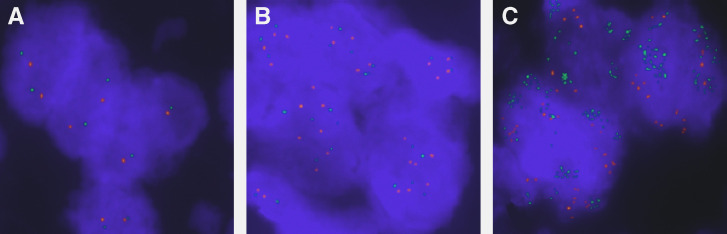
Representative fluorescent *in situ* hybridisation (*FISH*) images. (**A**) A tumour with diploid genotype showing most tumour cell nuclei containing two green signals (h*TERT*, 5p15.33) and two red signals (control locus, 5q31). (**B**) A polysomy tumour showing several signals of both the h*TERT* and 5q; (**C**) Tumour with high-level amplification with their nuclei containing 10–30 h*TERT* signals and two or more 5q signals.

Fluorescence *in situ* hybridisation was performed on 59 tumours that had been studied for h*TERT* gene copy by qPCR (Figure 3). The ratios between the h*TERT* (green probe) and 5q13 reference gene locus *D5S89* (red probe) signals were significantly correlated with the qPCR ratios of h*TERT*/*PIK3R1* gene content (Spearman correlation coefficient *r*=0.43, *P*=0.0006). A better correlation was found for ADC (*r*=0.61, *P*=0.0003) than for SQCC (*r*=0.34, *P*=0.086). hTERT gene amplification by FISH was found in 73% (43 out of 59) of tumours and there were significant correlations between qPCR and FISH results for all tumours (*P*=0.008, Table 4) and for ADC (*P*=0.004). Although patients with high h*TERT* gene copy number (high polysomy and amplification) by FISH were more likely to experience early recurrence compared to those with lower gene copy numbers (low polysomy, trisomy or disomy), the difference was not statistically significant (HR 1.51, 95% CI 0.61–3.76, *P*=0.37).

### hTERT mRNA expression

Reverse transcription–qPCR did not detect h*TERT* mRNA expression in several non-neoplastic lung samples; therefore, the expression level of each sample was arbitrarily represented relative to the median of the entire data set. We used the mean+2 s.d. of the non-neoplastic lung expression levels as the cutoff to dichotomise tumours into h*TERT* normal expression and overexpression groups. Overexpression occurred in 72% (94 out of 130) of NSCLC, but was significantly more frequent in SQCC (87%) compared to ADC (68%) (Figure 1B and Table 2). Overexpression was also associated with higher tumour stages (Table 2). Among tumours with expression data, hTERT gene copy results by qPCR were also available for 67 cases (Table 5). Overexpression correlated with amplification (*P*=0.03) but only among the ADC (*P*=0.05). There were only 54 tumours with both FISH and expression results; high h*TERT* gene copy by FISH was not correlated with mRNA overexpression (data not shown).

Kaplan–Meier estimation revealed only trends for association between h*TERT* mRNA overexpression with recurrence-free survival or overall survival (log rank *P*=0.24 and *P*=0.13, respectively, Figure 2C and D), but it was significant for reduced overall survival at the 3-year follow-up time (log rank *P*=0.03, Table 3). Cox proportional hazards regression also showed that overexpression was an independent prognostic marker for overall survival at 3-year follow-up (HR 2.29, 95% CI 1.06–4.96, *P*=0.04) after adjusting for age, tumour stage and differentiation.

## DISCUSSION

We have evaluated the clinical and pathological significance of h*TERT* gene amplification and mRNA overexpression in NSCLC patients who were treated primarily by surgical resection. The h*TERT* gene amplification occurred in 57% of NSCLC, but this was more common among ADC (75%) than SQCC (35%). Among ADC, *hTERT* mRNA overexpression was significantly correlated with gene amplification (*P*=0.05). However, 87% (33 of 38) of SQCC also showed overexpression. These findings suggest that amplification is responsible for h*TERT* mRNA overexpression in a majority of ADC, while epigenetic factors at the transcriptional or post-transcriptional levels significantly affect h*TERT* expression. Most importantly, we have demonstrated that h*TERT* amplification is an independent prognostic marker for shorter recurrence-free survival in NSCLC patients.

Although many studies have examined the prognostic significance of h*TERT* mRNA/protein expression or activity in NSCLC (Table 6), to our knowledge, this is the first study that examined the prognostic value of h*TERT* gene amplification in lung cancer patients. The h*TERT* gene amplification was common in cell lines and primary tumours of lung, cervix, breast and in neuroblastoma (Zhang *et al*, 2000). Using ⩾5 copies of h*TERT* gene copy per nucleus in at least 20% of the cells to define amplification, Zhang *et al* (2000) reported h*TERT* amplification in 38% (eight out of 21) of lung carcinomas. Using qPCR that defines amplification as tumours with h*TERT* gene content greater than that of *PIK3R1* (5q13.1), we found amplification in 57% of NSCLC. We also found a significant concordance between h*TERT* gene copy number assayed by qPCR and FISH (Spearman correlation coefficient *r*=0.43, *P*=0.0006), indicating that qPCR may serve as an alternative method to assay amplification.

There are some differences in the results of qPCR and FISH analyses on tumours. The qPCR assay cannot, whereas FISH can distinguish balanced copy gains (trisomy or polysomy) from diploid. To identify the former by qPCR, multiple reference genes or sequences on other chromosomes also need to be measured. The qPCR results also reflect the average gene copy number changes in DNA derived from thousands of microdissected tumour cells, whereas only a relatively small fraction (50 to few hundreds) of tumour cells are selected for FISH scoring. The poorer correlation between qPCR and FISH in SQCC compared to ADC could be due to the tendency of FISH to score tumour giant cells (cells with multilobated nuclei or multiple fused nuclei) that commonly contained multiple (5–10) copies of both h*TERT* and 5q reference loci; such giant tumour cells are more common in SQCC compared to ADC. Their scores may skew the assessment of gene copy number and introduce a higher variability in copy number estimation. Thus, the inability of our FISH studies to predict early recurrence of patients with h*TERT* gene amplification could result from: (1) smaller number of patients studied by FISH compared to qPCR, (2) cellular heterogeneity that skewed the overall FISH score due to recently acquired amplification in a discrete region within the tumour and (3) inconsistencies in FISH signal scoring criteria associated with polyploid nuclei and sectioning artefacts. Amplification has been defined differently in various FISH studies, such as ⩾3 (Murnane and Sabatier, 2004) or ⩾5 (Zhang *et al*, 2000) gene copies in at least 20% of the cells, or presence of tight gene clusters and a ratio of gene to chromosome of ⩾2 or ⩾15 copies of gene per cell in ⩾10% of analysed cells (Cappuzzo *et al*, 2005).

The reported frequencies of h*TERT* mRNA or protein overexpression in NSCLC ranged from 33 to 94%. In general, RT–qPCR data tended to demonstrate a higher overexpression rate compared to immunohistochemistry or mRNA *in situ* hybridization (mISH). Similar to our finding, most RT–qPCR studies but not other methods have also detected trace h*TERT* mRNA expression in normal lung tissues. We also found a correlation between h*TERT* amplification and overexpression, but mainly among the ADC.

The prognostic significance of h*TERT* expression or activity in NSCLC remains controversial (Table 6). Such an association has been reported in mISH (Kumaki *et al*, 2001; Wang *et al*, 2002; Fujita *et al*, 2003; Lantuejoul *et al*, 2004) and immunohistochemistry studies (Kumaki *et al*, 2001; Toomey *et al*, 2001; Lantuejoul *et al*, 2004). In contrast, the prognostic value of h*TERT* mRNA expression assayed by RT–qPCR has been inconsistent (Komiya *et al*, 2000; Hara *et al*, 2001; Marchetti *et al*, 2002; Wu *et al*, 2003; Hsu *et al*, 2004). Some studies have associated overexpression with poor prognosis (Komiya *et al*, 2000; Hara *et al*, 2001; Marchetti *et al*, 2002; Hsu *et al*, 2004), while others have failed to do so (Wu *et al*, 2003). One possible explanation is that h*TERT* mRNA has six known splice variants, but only the full-length transcript is functional. The qPCR probes designed in these studies did not distinguish between these variants. Although full-length mRNA level is usually proportional to the other variants (Fujiwara *et al*, 2004), it may range from 5 to 54% (Yi *et al*, 2001; Fujiwara *et al*, 2004). However, our finding that h*TERT* overexpression was predictive of poorer overall survival at 3 years is in agreement with a majority of these studies.

In conclusion, we have for the first time provided evidence that h*TERT* gene amplification or high copy number could be a marker for poorer prognosis in early-stage NSCLC patients, perhaps even more reliably than *hTERT* overexpression. While the clinical application of our findings requires more extensive retrospective and prospective validations in additional and larger cohorts of patients, further studies to evaluate the prognostic significance of h*TERT* by FISH is also warranted. In such case, a more refined FISH scoring system with reproducible criteria to identify clinically and biologically valid h*TERT* gene amplification would need to be developed. Since h*TERT* reactivation is a mechanism for cancer cells to avoid senescence (Shay and Roninson, 2004) and the latter could be induced by chemotherapy, the predictive value of h*TERT* amplification for benefit to adjuvant chemotherapy also needs evaluation (Winton *et al*, 2005). Recently, telomerase has been intensively studied as a target for novel cancer gene therapy and therapeutics (reviewed in Shay and Wright, 2002; Keith *et al*, 2004). Our finding that different types of NSCLC may alternately regulate h*TERT* overexpression suggests that patients with h*TERT* amplification could have different responses to telomerase-based therapies. The possible differential role of h*TERT* gene dosage in the diagnosis and treatment of lung cancer patients should be further investigated.

## Figures and Tables

**Table 1:** Clinical–pathological characteristics of patients in various molecular studies

	**Gene copy by qPCR (*n*=81) (%)**	**Gene copy by FISH (*n*=59) (%)**	**mRNA expression (*n*=130) (%)**	***P*- value**
*Age*				
Mean	69.0	69.9	68.8	0.73
Range	(43.7–85.4)	(43.7–85.4)	(43.7–85.4)	
				
*Gender*				
Male	50 (61)	35 (59)	74 (57)	0.79
Female	31 (39)	24 (41)	56 (43)	
				
*Smoking history*				
Smoker	59 (73)	41 (69)	92 (71)	0.99
Nonsmoker	19 (23)	16 (27)	33 (25)	
Unknown	3 (4)	2 (4)	5 (4)	
				
*Pathological stage*				
Stage I	52(64)	42 (71)	91 (70)	0.90
Stage II	21 (26)	12 (20)	28 (22)	
Stage III	8 (10)	5 (8)	11 (8)	
				
*T stage*				
T1	24 (30)	20 (34)	47 (36)	0.62
T2	54 (66)	37 (63)	82 (63)	
T3	3 (4)	2 (3)	1 (1)	
				
*N stage*				
N0	53 (65)	43 (73)	90 (69)	0.92
N1	20 (25)	11 (19)	28 (22)	
N2	8 (10)	5 (8)	12 (9)	
				
*Histology*				
ADC	40 (49)	32 (54)	83 (64)	0.03
SQCC	37 (46)	26 (44)	38 (29)	
Other	4 (5)	1 (2)	9 (7)	
				
*Differentiation grade*				
WD	20 (25)	16 (27)	33 (25)	0.98
MD	30 (37)	19 (32)	44 (34)	
PD	31 (38)	24 (41)	53 (41)	

The *P*-values were calculated using the *χ*^2^ test.

ADC=adenocarcinoma; SQCC=squamous cell carcinoma; WD=well differentiated; MD=moderately differentiated; PD=poorly differentiated; qPCR=quantitative polymerase chain reaction; FISH=fluorescence *in situ* hybridisation.

**Table 2:** h*TERT* copy number, mRNA expression and pathological factors

	**Gene copy**			**mRNA expression**		
	**Nonamplified (*n*=35) (%)**	**Amplified (*n*=46) (%)**	***P*-value**	**Normal expression (*n*=36) (%)**	**Overexpression (*n*=94) (%)**	***P*-value**
*Pathological stage*						
I	26 (74)	26 (57)	0.11	28 (78)	62 (66)	0.02
II	5 (14)	16 (35)		2 (6)	26 (28)	
III	4 (12)	4 (8)		6 (16)	6 (6)	
						
*T stage*						
T1	12 (34)	12 (26)	0.70	16 (44)	31 (33)	0.11
T2	22 (63)	32 (69)		19 (53)	63 (67)	
T3	1 (3)	2 (6)		1 (3)		
						
*N stage*						
N0	26 (74)	27 (59)	0.17	28 (78)	62 (66)	0.01
N1	5 (14)	15 (33)		2 (6)	26 (28)	
N2	4 (12)	4 (8)		6 (16)	6 (6)	
						
*Histology*						
ADC	10 (29)	30 (65)	<0.001	27 (75)	56 (60)	0.03
SQCC	24 (69)	13 (28)		5 (14)	33 (35)	
Other	1 (2)	3 (7)		4 (11)	5 (5)	
						
*Differentiation grade*						
WD	9 (26)	11 (24)	0.98	8 (22)	25 (27)	0.28
MD	13 (37)	17 (37)		16 (45)	28 (30)	
PD	13 (37)	18 (39)		12 (33)	41 (43)	

*P*-values were calculated by using *χ*^2^ test.

ADC=adenocarcinoma; SQCC=squamous cell carcinoma; WD=well differentiated; MD=moderately differentiated; PD=poorly differentiated; qPCR=quantitative polymerase chain reaction; FISH=fluorescence *in situ* hybridisation.

**Table 3:** Univariate and multivariate survival analyses at 3-year follow-up

	**Univariate**			**Multivariate** *		
	**HR**	**95% CI**	***P***	**HR**	**95% CI**	***P***
*Recurrence-free survival*						
Amplification	2.96	1.27–6.90	0.01	2.97	1.26–6.99	0.01
Overexpression	1.82	0.89–3.75	0.14	2.07	0.94–4.27	0.11
						
*Overall survival*						
Amplification	2.04	0.89–4.66	0.09	1.97	0.85–4.55	0.13
Overexpression	2.00	0.94–4.27	0.03	2.29	1.06–4.96	0.04

* Multivariate analyses adjusted for stage, differentiation grade and age.

HR=hazard ratio; CI=confidence interval.

**Table 4:** Correlation between gene copy by qPCR and by FISH

**Gene copy changes**	**FISH**		
**qPCR**	**Nonamplified**	**Amplified^a^**	***P*-value^b^**
*All*	*22*	*37*	
Nonamplified	13	9^c^	0.008
Amplified	9	28	
			
*Adenocarcinoma*			
Nonamplified	6	1	0.004
Amplified	5	18	
			
*Squamous cell carcinoma*			
Nonamplified	7	7	0.5
Amplified	4	8	

a Amplified tumours were those showing presence of tight h*TERT* gene clusters or h*TERT* to chromosome ratio of ⩾2, or ⩾15 copies per cell in ⩾10% of tumour cells, as defined by Cappuzzo *et al* (2005).

b *P*-values calculated using the two-sided Fisher's exact test.

c One case was a large-cell carcinoma.

qPCR=quantitative polymerase chain reaction; FISH=fluorescence *in situ* hybridisation.

**Table 5:** Correlation between gene copy increases by qPCR and h*TERT* mRNA expression

	**Gene copy changes**		
	**Nonamplified**	**Amplified**	***P***-**value^a^**
*All*	*27*	*40*	
Normal expression	9^b^	4	0.03
Overexpression	18	36	
			
*Adenocarcinoma*			
Normal expression	4	4	0.05
Overexpression	4	24	
			
*Squamous cell carcinoma*			
Normal expression	4	0	0.12
Overexpression	14	12	

a *P*-values calculated using the two-sided Fisher's exact test.

b One case was a large-cell carcinoma.

qPCR=quantitative polymerase chain reaction.

**Table 6:** Previous reports on the prognostic significance of telomerase

		**Prognostic significance**		
	**Case**	**Activity**	**hTERT expression (assay)^a^**	**TRFLR^b^**
Albanell *et al* (1997)	99	No	—	No
Taga *et al* (1999)	103	Yes	—	—
Komiya *et al* (2000)	68	—	Yes (RT–qPCR)	—
Hirashima *et al* (2000)	72	No	—	Yes
Arinaga *et al* (2000)	92	—	No (RT–qPCR)	—
Kumaki *et al* (2001)	115	Yes	No (mISH, IHC)	—
Hara *et al* (2001)	62	—	Yes (RT–semi-qPCR)	—
Toomey *et al* (2001)	115	—	No (IHC)	—
Wang *et al* (2002)	153	—	Yes (mISH)	—
Marchetti *et al* (2002)	90	Yes	YES (RT–qPCR)	—
Wu *et al* (2003)	56	Yes	No (RT–qPCR)	—
Fujita *et al* (2003)	146	—	Yes (mISH)	—
Hsu *et al* (2004)	48	No	—	Yes
Lantuejoul *et al* (2004)	122	—	Yes (mISH, IHC)	—
Lu *et al* (2004)	94	—	No (mISH)	—

a —=not studied; RT–qPCR=reverse transcription–quantitative polymerase chain reaction; mISH=mRNA *in situ* hybridisation; IHC=immunohistochemistry.

b Telomere terminal restriction fragment length ratio (tumour *vs* normal).
